# Conditional Deletion of Ferritin H in Mice Reduces B and T Lymphocyte Populations

**DOI:** 10.1371/journal.pone.0089270

**Published:** 2014-02-21

**Authors:** Liviu Vanoaica, Larry Richman, Maike Jaworski, Deepak Darshan, Sanjiv A. Luther, Lukas C. Kühn

**Affiliations:** 1 Swiss Institute for Experimental Cancer Research (ISREC), Sciences de la Vie (SV), Ecole Polytechnique Fédérale de Lausanne (EPFL), Lausanne, Switzerland; 2 Department of Biochemistry, University of Lausanne, Epalinges, Switzerland; 3 Queensland Institute of Medical Research, Royal Brisbane Hospital, Brisbane, Australia; University of Alberta, Canada

## Abstract

The immune system and iron availability are intimately linked as appropriate iron supply is needed for cell proliferation, while excess iron, as observed in hemochromatosis, may reduce subsets of lymphocytes. We have tested the effects of a ferritin H gene deletion on lymphocytes. Mx-Cre mediated conditional deletion of ferritin H in bone marrow reduced the number of mature B cells and peripheral T cells in all lymphoid organs. FACS analysis showed an increase in the labile iron pool, enhanced reactive oxygen species formation and mitochondrial depolarization. The findings were confirmed by a B-cell specific deletion using *Fth^lox/lox^*; CD19-Cre mice. Mature B cells were strongly under-represented in bone marrow and spleen of the deleted mice, whereas pre-B and immature B cells were not affected. Bone marrow B cells showed increased proliferation as judged by the number of cells in S and G2/M phase as well as BrdU incorporation. Upon *in vitro* culture with B-cell activating factor of the tumor necrosis factor family (BAFF), ferritin H-deleted spleen B cells showed lower survival rates than wild type cells. This was partially reversed with iron-chelator deferiprone. The loss of T cells was also confirmed by a T cell-specific deletion in *Fth^lox/lox^*;CD4-Cre mice. Our data show that ferritin H is required for B and T cell survival by actively reducing the labile iron pool. They further suggest that natural B and T cell maturation is influenced by intracellular iron levels and possibly deregulated in iron excess or deprivation.

## Introduction

All cells need iron for the *de novo* synthesis of heme-, iron-, or iron-sulfur cluster containing proteins. This requires a cytoplasmic “labile iron pool” (LIP) of about 1 µM divalent iron [1]. The LIP is a transit pool at the cross-road of import and export of iron across the plasma membrane, of iron transport to mitochondria, and of iron deposition or release to and from the storage compartment of ferritin. The LIP can be measured by the quenching of the fluorescent probe calcein or by reversing the quenching with iron-specific chelators [2]. Besides of being essential, divalent iron in conjunction with side-products of mitochondrial respiration, hydrogen peroxide and superoxide ion, catalyzes the formation of radicals, collectively called “reactive oxygen species” (ROS). To escape damage by ROS, cellular defense mechanisms include a permanent feedback control over the LIP. In particular the syntheses of transferrin receptor 1 (TfR1), which functions in iron uptake, and ferritin H and L, which form the iron storage compartment to capture excess cytoplasmic iron, are adjusted to the LIP. This is achieved by the iron regulatory proteins 1 and 2, which bind to iron responsive elements on the respective mRNAs to control RNA translation and stability [3], [4], [5], [6]. As a result, the steady state level of the LIP is maintained within a range that prevents damage, but ensures sufficient iron supply for biosynthetic pathways in the cytoplasm and mitochondria.

Ferritin is an assembled hollow protein shell composed of 24 subunits of ferritin H (Fth) and L at variable stoichiometry that store iron [7]. Storage of iron into ferritin requires the ferroxidase activity of Fth protein [8], [9]. Ferritin is thought to have a role in providing iron stores to the cytoplasm when cells have to cope with cell divisions, such as in embryos or during the immune response [10], [11], to ensure de novo synthesis of iron-containing proteins. On the other hand, the role of Fth as a regulator of the LIP has been the subject of several investigations in cell culture [1], [9], [12], [13]. Reduction of Fth expression by antisense mRNA, siRNA, or genetic ablation, increased the LIP and initiated ROS production. Although Fth synthesis is mainly translationally regulated, Fth gene transcription can also be induced by cytokines, such as TNFα, through NF-κB activation [12], [14]. TNFα primarily activates the MAPK pathway ending in JNK activation and ROS accumulation, which provokes ultimately caspase-dependent cell death. The ROS-dependent death is counteracted by parallel activation of NF-κB. The Fth gene was revealed as an essential NF-κB target with an anti-apoptotic effect similar to iron chelation or ROS inhibitors [12]. Only Fth with an active ferroxidase activity protected cells, indicating that TNFα-induced ROS accumulation involves the LIP and sequestering of iron into ferritin is required to prevent cell death [12].

During their development, B and T cells undergo various steps of cell proliferation, as well as positive and negative selection to generate the immune repertoire [15], [16]. The MAPK and JNK pathways activated by Toll-like or T cell receptors contribute to negative selection by apoptosis, while NF-κB promotes cell survival [17], [18]. Thus, as in 3T3 cell cultures, NF-κB-mediated Fth synthesis is potentially important to prevent lymphocyte death by blocking ROS formation [12].

There exist various reports that a deregulation of cellular iron supply may perturb the immune system. Cell proliferation requires iron [19] and intracellular iron stores in ferritin are thought to sustain mitogen-stimulated proliferation of immune cells [10], . Iron-deficiency reduces T-lymphocyte numbers and impairs natural killer cell activity [20]. Similarly, loss of iron uptake in *TfR1* deleted mice impairs T-cell development at an early CD4^−^8^−^3^−^ stage and reduces mature B-cell numbers [21]. Patients with iron-overload in β-thalassemia major have decreased CD4^+^ and increased CD8^+^ T cells [22], while idiopathic hemochromatosis patients show a trend to lower CD8^+^ T cells depending on the HLA haplotype [23], [24], [25]. It was therefore of interest to test whether deletion of ferritin iron stores would alter lymphocyte proliferation or survival.

We have analyzed the conditional deletion of Fth by the interferon regulated Mx-Cre allele in mice. Bone marrow and peripheral lymphocyte compartments showed a partial loss of mature B and T cells. We have characterized the B and T cell subsets with respect to iron-mediated alterations and found an increased LIP and mitochondrial depolarization as hallmarks correlating with the loss of lymphocytes. Short-term cultures of splenic B cells with B-cell activating factor of the tumor necrosis factor family (BAFF) indicated that Fth was necessary for the survival of mature B cells. These findings were confirmed *in vivo* with B- and T-cell specific Fth deletions. The results highlight that ferritin controls the LIP and is required to prevent ROS formation and cell death.

## Materials and Methods

### Animals

Mice were maintained at the EPFL animal facility under pathogen free conditions and housed in individually ventilated cages. Animal experimentation was performed according to protocols approved by the Swiss Veterinary Office, authorization 1802. *Fth^lox/lox^* control mice in C57BL/6J background [9] were crossed with Mx-Cre transgenic mice [26] to obtain Mx-Cre;Fth*^lox/lox^* mutant mice. To study the conditional Fth deletion in bone marrow, thymus and spleen, 10-week old control and mutant mice were injected i.p. with polyinosinic-polycytidylic acid (poly-IC) (InvivoGen, San Diego, CA) every 2–3 days, either 5 times with 0.1 mg/kg or twice with 1.0 mg/kg. *Fth^lox/lox^* and wild-type *Fth^+/+^* mice were crossed to CD4-Cre mice (Taconic Transgenic NIAID Exchange no 4196F; B6.Cg-Tg(CD4-cre)1Cwi N9) [27] to generate CD4-Cre;Fth*^lox/lox^* and CD4-Cre;*Fth^+/+^* mice. *Fth^lox/lox^* and *Fth^+/+^* mice were crossed to CD19-Cre mice (Jackson Laboratory no 001426; C.Cg-*Cd19^tm1(cre)Cgn^ Igh^b^*/J) [28] to obtain CD19-Cre;Fth*^lox/lox^* and CD19-Cre;*Fth^+/+^* mice. These mice were further crossed to Rosa26-EYFP transgenic mice (Jackson Laboratory no 006148; B6.129X1-*Gt(ROSA)26Sor^tm1(EYFP)Cos^*/J) [29] to generate CD19-Cre; Fth*^lox/lox^*;EYFP*^lox/lox^* (mutant) and CD19-Cre; *Fth^+/+^*;EYFP^lox/lox^ (control) mice. Presence or absence of Cre and loxP sites in the Fth allele were determined by PCR as described previously [9]. The Rosa26-EYFP allele genotyping was according to the Jackson Laboratory protocol database. Fth mRNA was quantified by Taqman real-time PCR and normalized to mouse acidic ribosomal protein 0 mRNA as described [9]. The average mRNA level in Fth*^lox/lox^* mice was set as 100%. The level in *Fth* ^Δ/Δ^ mice and standard deviations were normalized to the level in Fth*^lox/lox^* mice. For quantification of the genomic DNA deletion, the forward primer 5′CATCAACCGCCAGATCAAC3’ was in the deleted Fth gene 1st exon, and for normalization it was outside the deleted region in the *Fth* 1^st^ intron, at 5′ TTCAAGCTTGGATCCGTTTA3’. Both forward primers were used in conjunction with the probe 5′famTGGCTCCTGCATAGATCAGGCATGT3’tamra and reverse primer 5′GCCAGGCTGACAGTCGTACT3’ in the *Fth* 1^st^ intron. The TACI:Fc mouse strain was a kind gift from P. Schneider. Mice were sacrificed by carbon dioxide inhalation.

### Antibodies

Primary anti-mouse antibodies to surface markers were: PerCP-Cy5.5-conjugated anti-CD3ε (145-2C11, eBioscience, San Diego, CA), Alexa647-conjugated anti-CD4 (H129.19.6, BD Pharmingen, San Diego, CA), Pacific Blue-conjugated anti-CD4 (GK1.5, BioLegend, San Diego, CA), PE-Cy7- and Alexa Fluor 700-conjugated anti-CD8α (53-6.7, eBioscience), biotin-conjugated anti-CD11b (M1/70, BioLegend), PE-Cy5-conjugated anti-CD19 (6D5, eBioscience), biotin-conjugated anti-CD25 (PC61.5, eBioscience), PE- and biotin-conjugated anti-CD43 (eBioR2/60, eBioscience), FITC-conjugated anti-CD44 (KM81, Immunotools, Friesoythe, Germany), PE-Cy7-conjugated anti-CD45 (30-F11, BioLegend), PE-Cy7-conjugated anti-CD45R (B220) (RA3-6B2, eBioscience), APC-conjugated anti-CD93 (AA4.1, eBioscience), FITC-conjugated anti-Gr1 (RB6-8C5, BioLegend), PE-Cy5-conjugated anti-Ter119 (TER-119, eBioscience), biotin-conjugated anti-IgM (LO-MM, Invitrogen, Carlsbad, CA), FITC- conjugated anti IgD (11-26C, eBioscience). PeCy7-conjugated anti-CD24 (M1769, Biolegend) was used in conjunction with eFluor450NC-anti-CD4 (GK1.5, eBioscience) and APC-labelled anti-CD8α (53-6.7, BioLegend). Secondary antibodies were APC-Cy7- or eFluor450-conjugated streptavidin (eBioscience).

### Flow Cytometry

Cells were acquired on a FACScan®, FACSCanto® or LSRII® flow cytometer (Becton Dickinson, Franklin Lakes, NJ) and analyzed with FlowJo software (TreeStar, Ashland, OR). Cells from bone marrow, spleen, thymus and lymph nodes were isolated and passed through a 40 µm mesh, washed and resuspended in RPMI-1640 medium, 10% FCS. Isolated cells were resuspended in 10 ml DMEM, 2% FCS (with Pen/Strep and HEPES diluted 1/100) on ice. Living cells were counted using trypan blue exclusion. Antibody staining was performed with 1 to 1.5×10^6^ cells in 96-well V-bottom plates (Falcon). Cells were blocked with 1% normal mouse serum and antibodies added in 25 µl PBS containing 2% FCS, 2 mM EDTA and 1% NaN_3_. Cells were washed twice with 200 µl of this buffer after each step.

### Splenic B-cell Survival Assay

Splenocytes were filtered through a 40 µm-mesh Falcon cell strainer (Becton-Dickinson) and centrifuged in 8 ml complete RPMI 1640 medium (Invitrogen), 10% FCS, with antibiotics penicillin-streptomycin-neomycin (PSN) (Invitrogen), 50 µM 2-mercaptoethanol over a 5 ml cushion of Ficoll-Paque (GE Healthcare, Pollards Wood, UK) at 700×*g* for 30 min at 22°C. After washing in medium, the lymphocyte layer was resuspended at 10^7^ cells per 100 µl MACS buffer (PBS, 0.5% BSA, 2 mM EDTA) containing 10% anti-CD19 antibody beads (Miltenyi, Bergisch Gladbach, Germany) and incubated on ice for 15 min before separating on MACS LS column (Miltenyi). CD19-positive B cells were cultured in 96-well round bottom plate at 3×10^5^ cell per well in 200 µl complete media for 1–3 days at 37°C and 5% CO_2_ before assaying for viability by the cytometric scatter profile using a CyAn cytometer. Survival assays were carried out in the presence or absence of 20 ng/ml BAFF in the His-tagged, recombinant 60-mer configuration [30] (courtesy by P. Schneider). Where indicated, the iron chelator 3-hydroxy-1, 2-dimethyl-4(1H)-pyridone (deferiprone) (Sigma-Aldrich) was added to cell cultures at 300 µM for 24 h.

### Cell Cycle Analysis

For BrdU analysis, mice were injected i.p. with BrdU (9 mg/ml, 10 µl/g of mouse weight) twelve hours prior to analysis and subsequently kept on BrdU-containing water (1 mg/ml). Mice were sacrificed and bone marrow cells were isolated. By suspension in red blood cell lysis buffer (17 mM Tris-base, 139 mM NH_4_Cl, pH 7.2) most erythrocytes were eliminated. B lymphocytes were sorted into EYFP-positive and -negative populations on an Aria flow cytometer. A commercially available kit (BD Pharmingen, BD Biosciences, San Diego, CA) was used to fix, permeabilize and stain cells against BrdU, according to the manufacturer’s instructions.

### Detection of ROS

Dihydroethidium (Sigma-Aldrich, St. Louis, MO) was prepared as a 10 mM stock solution in DMSO. After antibody-staining of surface markers and washing in PBS, 2% FCS, cells were incubated in 250 µl of 10 µM dihydroethidium in HBSS buffer (Invitrogen, Carlsbad, CA) for 35 min at 37°C. Cells were analyzed on a LSRII flow cytometer (Becton-Dickinson, Franklin Lake, NJ) immediately after incubation, without washing.

### Detection of Mitochondrial Depolarization and LIP by Flow Cytometry

Tetramethyl rhodamine methyl ester (TMRM) (Sigma-Aldrich) (5 mM) and calcein AM (Biotium, Hayward CA) (1 mM) were dissolved in DMSO. Following antibody staining and washing in PBS, 2% FCS, cells were washed and stained in HBSS with 250 nM TMRM and 4 nM calcein AM, either together or separately, for 40–60 min at 37°C. In some instances 0.5 µg/ml 7-aminoactinomycin D (7AAD) was added at room temperature shortly prior to analysis. Events were acquired directly without washing using a LSRII flow cytometer or a CyAn cytometer (Dako, Glostrup, Denmark). To test depolarization, the protonophore m-chlorophenylhydrazone (CCCP) (Sigma-Aldrich) was prepared as a 100 mM stock solution in DMSO and added to the staining solution at 100 µM. For the analysis of the LIP, deferiprone was prepared as a 50 mM stock solution in 1% NaCl and added to the staining solution at 300 µM.

### Statistics

Data are presented as average values ± SD. All FACS experiments were analyzed once for each mouse without replicates. Cell culture experiments were done in duplicates. Statistic significance was assessed by paired or unpaired Student’s t-tests or by single factor Anova in case of comparison of more than two series.

## Results

### Effects of Mx-Cre Mediated Fth Deletion on the Immune System

**Figure 1:**
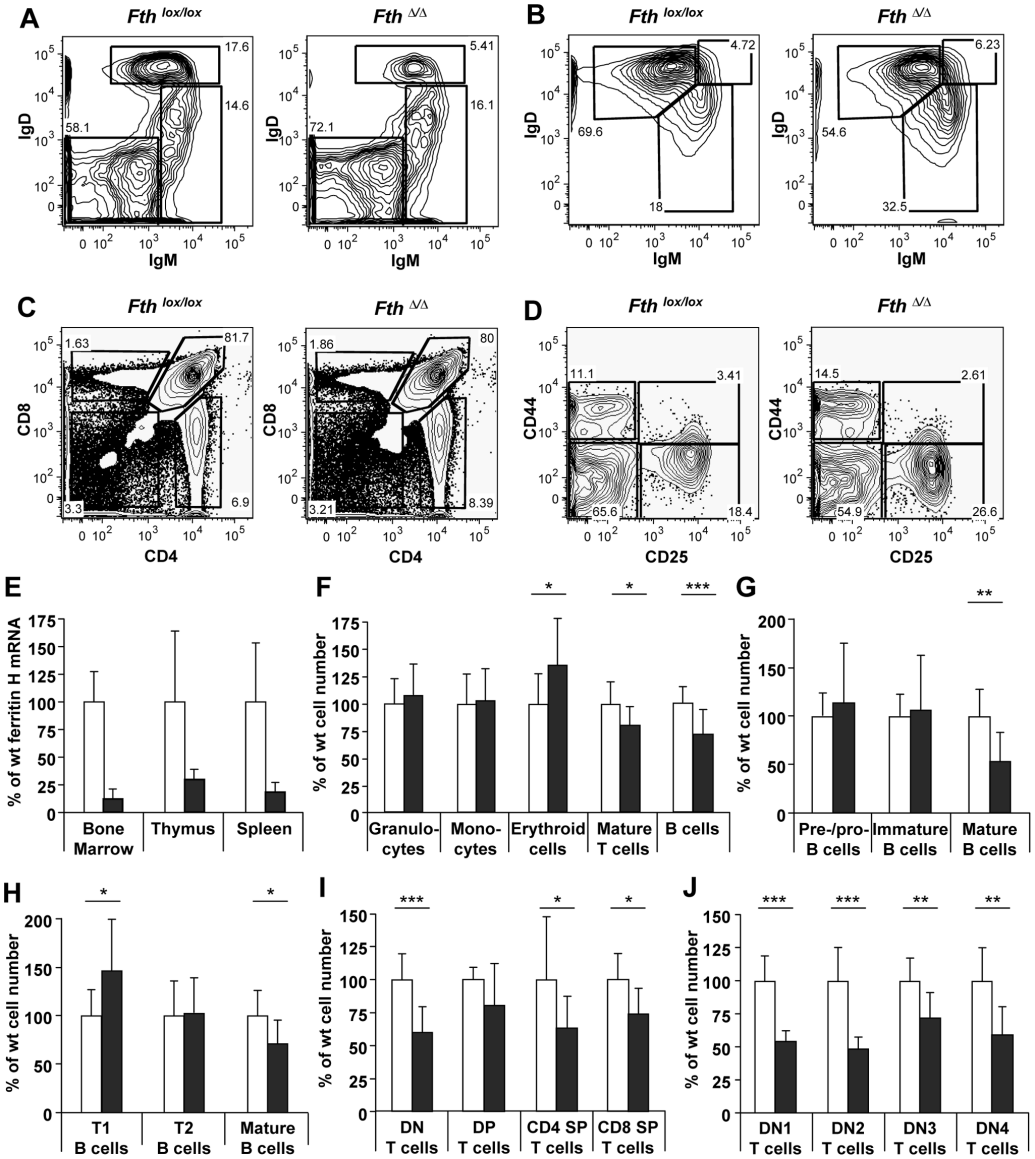
Fth deleted mice show reduced number of mature B and T cells. 10–18 week old Mx-Cre transgenic *Fth^lox/lox^* mice or non-transgenic *Fth^lox/lox^* mice were injected 5 times with poly-IC over 8 days and analyzed on day 30. Results for *Fth^lox/lox^* (*white*) or *Fth* ^Δ/Δ^ mice (*grey*) are shown as % of each cell population normalized to the average in *Fth^lox/lox^* mice (100%). **E.** Deletion efficiency of Fth mRNA measured in bone marrow, thymus and spleen (n = 9). **F–H.** Suspensions of bone marrow and spleen cells were stained with antibodies, analyzed by flow cytometry and plotted as numbers in experimental versus control mice (n = 8–9). **F.** Bone marrow subpopulations were identified as follows: granulocytes (Ter119^−^CD11b^+^GR1^high^), monocytes (Ter119^−^CD11b^+^GR1^low^), nucleated erythroid cells (Ter119^+^CD4^−^CD8^−^), T cells (CD4^+^ or CD8^+^) and B cells (CD19^+^CD45^+^; pool of precursor and mature B cells). **G.** Bone marrow B-cell populations (CD19^+^CD45^+^) were stained with relevant antibodies and gated into prepro−/pro-B cells (IgD^−^IgM^−^), pre−/immature B cells (IgD^−^μ^+^ or IgD^−^IgM^+^) and mature B cells (IgD^+^IgM^+^) as shown in panel **A**. **H.** Splenic B-cell populations (CD19^+^CD45^+^) were stained with relevant antibodies and gated into transitional (T)1 B cells (IgD^int^ IgM^hi^), T2 B cells (IgD^hi^ IgM^hi^), and mature B cells (IgD^hi^ IgM^int^) as shown in panel **B**. **I–J.** Suspensions of thymocytes were stained with antibodies, analyzed by flow cytometry and plotted as numbers in experimental versus control mice (n = 8–9). **I.** Analysis, of the four major thymocyte subpopulations: double-negative (DN; CD4^−^CD8^−^CD3^−^), double-positive (DP; CD4^+^CD8^+^), CD4 single positive (CD4 SP; CD4^+^CD8^−^) and CD8 single positive (CD8 SP; CD4^−^CD8^+^) as shown in panel **C**. % of each cell population was normalized to the average in *Fth^lox/lox^* mice. **J.** Analysis of the four earliest, double negative (DN) thymocyte subsets (CD4^−^CD8^−^CD3^−^): DN1 (CD44^+^CD25^−^), DN2 (CD44^+^CD25^+^), DN3 (CD44^−^CD25^+^) and DN4 (CD44^−^CD25^−^) as shown in panel **D.** Results are compiled of three independent experiments with each having 2–3 mice per group. ***p<0.0005; **p<0.005; *p<0.05.

To test whether the development and homeostasis of hematopoietic cell lineages depend on ferritin, we analyzed bone marrow, thymus and spleen 30 days after Mx-Cre induced Fth deletion. The deletion efficiency based on Fth mRNA was 88±8% in the bone marrow, 71±9% in thymus, and 82±7% in spleen (Fig. 1E), in the range of previous studies [26], [31], [32]. In the bone marrow of experimental versus control mice, no difference was found in the number of granulocytes and monocytes/macrophages, while the number of nucleated erythroid cells was increased, and that of mature T cells and B-lineage cells significantly decreased (Fig. 1F). Analysis of the developmental stages of B cells revealed a 50% reduction in mature B cells, while pre−/pro- and immature B cells were not affected (Fig. 1G). As B cells leave the bone marrow at the immature B cell stage to complete their maturation in the spleen [33], the number of the two transitional immature B cell populations (T1 and T2) as well as of mature B cells in the spleen was assessed. While T2 B-cell numbers were not altered, the number of T1 B cells was increased and that of mature B cells decreased (Fig. 1H). This finding is consistent with the reduction of mature B cells in the bone marrow.

To test whether Fth also plays a role in T lymphocyte development, the various thymocyte subsets were investigated. Thymocytes go through a double-negative stage before turning on the expression of CD4 and CD8. Once both genes are expressed, double-positive thymocytes are positively and negatively selected giving rise to mature single-positive T lymphocytes expressing either CD4 or CD8 [34], which may then migrate to the periphery and further mature [16]. In Mx-Cre transgenic *Fth* ^Δ/Δ^ mice the total thymocyte number was reduced on average by 20% relative to control mice, although the reduction in double-positive thymocyte numbers was not significant (Fig. 1I). Interestingly, the proportion of the four subsets was unaffected. TfR1 staining was not significantly altered (not shown). To investigate further the defect in early thymocyte development, double-negative (DN) thymocytes were stained for CD44 and CD25 to distinguish the four developmental stages from DN1 to DN4. The cell number of all four stages was reduced by 30–50% in experimental mice but the fraction of each stage did not change (Fig. 1J). Therefore, the thymic T cell development defect does not correlate with the phases of genetic recombination at the T cell receptor locus, its expression and cell proliferation.

### Fth Deletion Increases the Labile Iron Pool and Selects against Mature B Cells in the Bone Marrow

**Figure 2:**
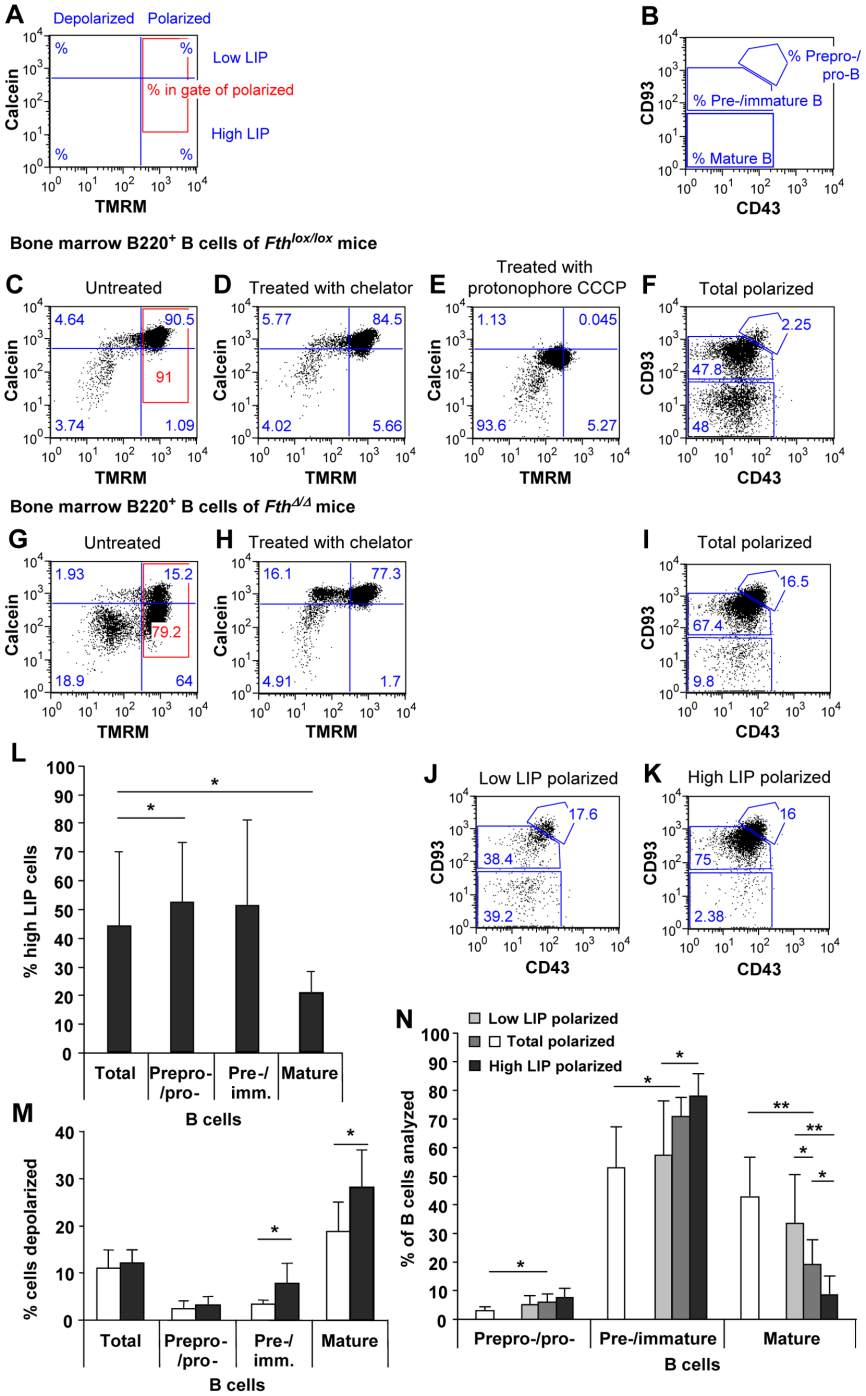
Increased LIP and mitochondrial depolarization in bone marrow B-cell populations of Fth deleted mice. B220^+^ B cells in bone marrow were selected with PE-Cy7-conjugated anti-B220 antibody. Cells were stained with TMRM to assess their mitochondrial polarization and with calcein AM for cell viability and LIP content. Using FACS gating (**A**), cells with polarized mitochondria (red zone) were analyzed with APC-conjugated anti-CD93 and PE-conjugated anti-CD43 to distinguish B-cell subsets (**B**). **C.** Typical FACS profile of total B cells of *Fth^lox/lox^* mice with high calcein staining indicating a low LIP. **D.** The number of low-LIP cells was virtually unaltered by the iron-chelator deferiprone. In contrast, most cells of *Fth* ^Δ*/*Δ^ mice showed low calcein staining and hence a high LIP (**G**) that was strongly reduced by deferiprone (**H**). TMRM showed a similar average staining in *Fth^lox/lox^* (**C**) and *Fth* ^Δ/Δ^ mice (**G**). However, the number of cells with depolarized mitochondria (low TMRM) was higher in *Fth* ^Δ/Δ^ mice correlating with a high LIP (**G**). **E.** Adding the protonophore CCCP depolarized mitochondria in all cells. **F.** B220^+^ B cells with polarized mitochondria of *Fth^lox/lox^* mice (red zone in **C**) were analyzed for B cell subsets. **I.** B220^+^ B cells with polarized mitochondria of *Fth* ^Δ/Δ^ mice (red zone in **G**) were analyzed for B cell subsets. **J–K.** The same cells were subdivided into cells with low and high LIP (above or below blue line in the red zone) to determine the subset composition of each fraction. **L.** Percent cells in each B-cell subset of *Fth* ^Δ/Δ^ mice that show a high LIP. Few high LIP cells (<10%) were detected in B-cell subsets of *Fth^lox/lox^* control mice, and are not reported. **M.** Percent cells with low TMRM fluorescence indicating mitochondrial depolarization in each B-cell subset of *Fth^lox/lox^* (*white*) and *Fth* ^Δ/Δ^ mice (*grey*). **N.** Graphical representation of all data gathered by **F** and **I–K**. B cells in each subset are expressed as % of cells analyzed in *Fth^lox/lox^* (*white*), *Fth* ^Δ/Δ^ mice (*medium grey*), or *Fth* ^Δ/Δ^ mice with low LIP (*light grey*) or high LIP (*dark grey*). Subsets for each color add up to 100%. Results are average values of 8 mice ± SD. ***p<0.0005; **p<0.005; *p<0.05.

To test the cause of the lymphocyte decline, bone marrow B cells were examined by flow cytometry with probes for the LIP and mitochondrial polarization in combination with surface marker antibodies. B220^+^ B cells of bone marrow were stained with calcein AM and trimethyl rhodamine methyl ester (TMRM) to define cells with low or high LIP and with polarized or depolarized mitochondria (Fig. 2A). The same cells were also characterized with respect to CD93 and CD43 antigen expression to distinguish three major subsets (Fig. 2B) [35]. Calcein, a FITC-fluorochrome, is quenched by binding cytoplasmic divalent iron, and used to detect differences in the LIP [36], [37]. Strong quenching and hence less calcein fluorescence is observed at high LIP. Reversion of the quenching by iron chelators, such as deferiprone, serves as a proof that the iron was indeed labile and accessible (Fig. 2D, 2H). Moreover, staining with the non-fluorescent calcein AM derivative distinguishes viable from dead lymphocytes. Dead cells lack the hydrolase activity necessary to remove the acetyl group from calcein AM and are negative for calcein fluorescence. Finally, the fluorescent probe TMRM is selectively taken up by polarized mitochondria due to the proton gradient. In the early stages of cell death, mitochondria depolarize and decrease in TMRM fluorescence after loss of the gradient [38], [39].

In the bone marrow of *Fth^lox/lox^* mice, an inconsistent number of B220^+^ B cells, usually less than 10%, had a high LIP (Fig. 2C). Most of these cells showed depolarized mitochondria and did not respond to iron chelation (Fig. 2D) indicating that they were primarily non viable. They were also permeable to 7-aminoactinomycin D (7AAD) (not shown). In contrast, in *Fth* ^Δ/Δ^ mice, up to 86% showed a high LIP (Fig. 2G). This strong quenching of calcein fluorescence was readily reversed by iron chelation (Fig. 2H). Thus, a high LIP fraction could only be reliably defined in the cell fraction with polarized mitochondria of *Fth* ^Δ/Δ^ mice (Fig. 2G). On average 44% of total B cells in *Fth* ^Δ/Δ^ mice had a high LIP (Fig. 2L), with a strong variability of 16–86% that correlated with the degree of mRNA deletion (n = 7, p<0.05). In the pre-B and immature B-cell subsets, the high LIP fraction was on average 52% in *Fth* ^Δ/Δ^ mice, whereas in mature B cells it was only 21% (Fig. 2L), suggesting the loss of mature B cells with high LIP in *Fth* ^Δ/Δ^ mice. These results were supported by those for mitochondrial depolarization (Fig. 2M). The mature B-cell subset showed the highest fraction of cells with depolarized mitochondria in *Fth* ^Δ/Δ^ mice suggesting that they were more readily damaged by high LIP. Most of the cells with high LIP in *Fth* ^Δ/Δ^ mice showed no mitochondrial depolarization in comparison to protonophore-induced depolarization (Fig. 2E). However, 15–20% of high LIP cells in *Fth* ^Δ/Δ^ had depolarized mitochondria (Fig. 2G) of which a substantial fraction was consistently unquenched by iron chelation (Fig. 2H). The prevalence of a high LIP and depolarization in *Fth* ^Δ/Δ^ cells suggests a causative relation between the high LIP and cell death. In addition, a selection against mature B cells was apparent in cells with polarized mitochondria (Fig. 2F,I,N). Mature B cells decreased from 43% in *Fth^lox/lox^* to 19% in *Fth* ^Δ/Δ^ mice with corresponding increases in the prepro−/pro- and pre−/immature B-cell subsets. Moreover, when only cells with polarized mitochondria and high LIP were analyzed, the frequency of mature B cells was 8% in *Fth* ^Δ/Δ^ mice (Fig. 2K,N).

### Selection against Early and Mature T Cell Stages in Fth Deleted High LIP Cells of the Thymus

**Figure 3:**
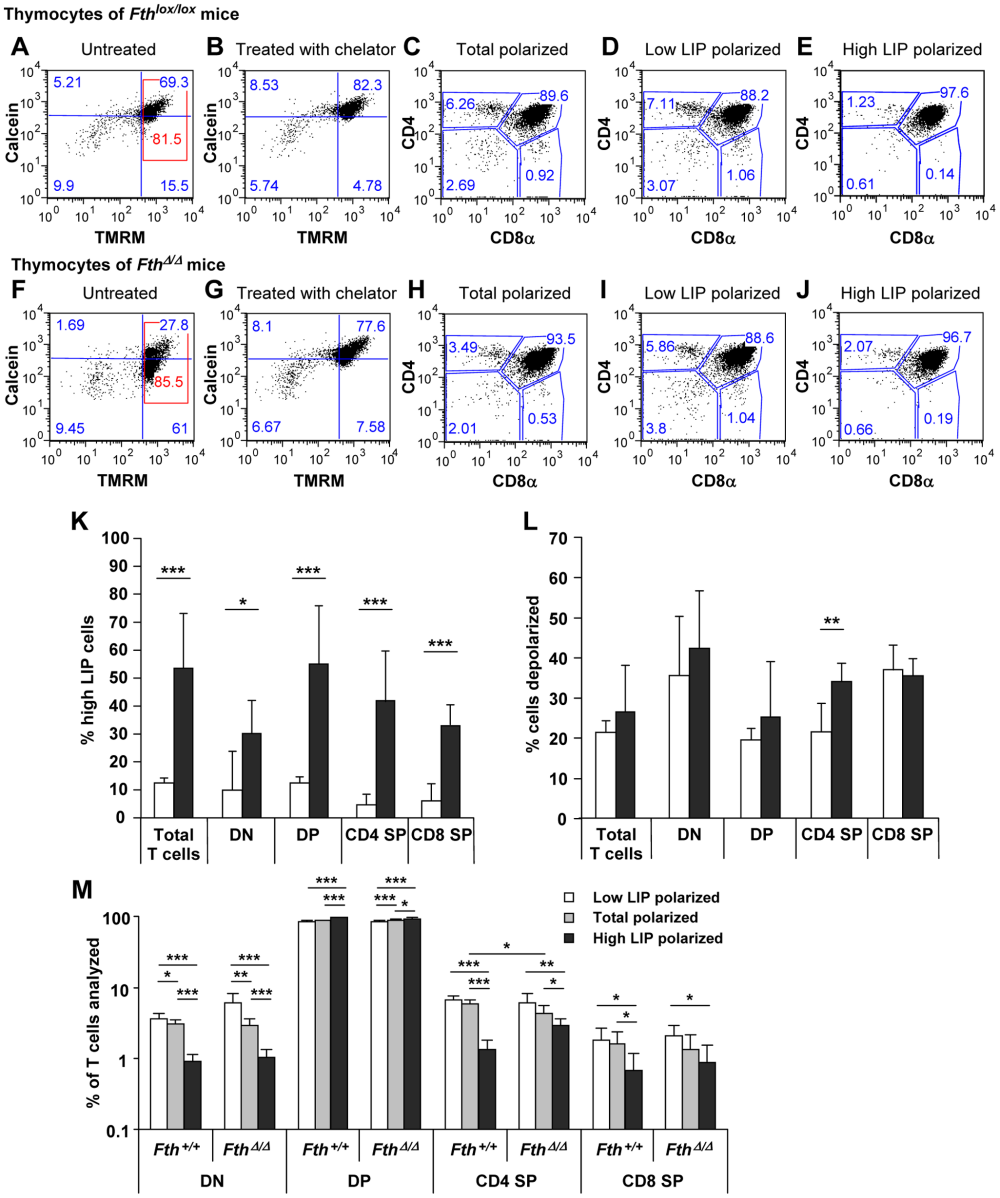
Increased LIP and mitochondrial depolarization in thymocytes of Fth deleted mice. Thymocytes were stained with Pacific Blue-conjugated anti-CD4 and Alexa Fluor 700-A conjugated anti-CD8α to analyze their state of T-cell differentiation, followed by TMRM for mitochondrial depolarization and calcein AM for cell viability and LIP content. FACS analysis was carried out on cells from *Fth^lox/lox^* (**A–E**) and *Fth* ^Δ/Δ^ mice (**F–J**). **C–E** and **H–J** show a representative FACS gating used to distinguish double-negative cells in lower left zone (DN; CD4^−/^CD8α^–^), double-positive cells in upper right zone (DP; CD4^+^/CD8α^+^), single-positive cells for CD4 in upper left zone (CD4 SP; CD4^+^/CD8α^–^), and single-positive cells for CD8α in lower right zone (CD8 SP; CD4^−/^CD8α^+^). Most T cells showed a high calcein staining in *Fth^lox/lox^* mice representing a low LIP (**A**). Only about 10% of cells with polarized mitochondria showed low calcein staining, which was unquenched by the iron chelator deferiprone (**B**). In contrast, in *Fth* ^Δ/Δ^ mice about 80% of cells with polarized mitochondria showed a low calcein staining representing a high LIP (**F**) that was unquenched by deferiprone (**G**). Double staining with mitochondrial depolarization marker TMRM showed a similar average staining in *Fthl^ox/lox^* and *Fth* ^Δ/Δ^ mice in spite of a very different LIP. Only a small fraction of cells showed depolarized mitochondria. Adding the protonophore CCCP depolarized mitochondria in all cells (not shown). For the analysis of T cell subsets (**C–E** and **H–J**), only cells with polarized mitochondria (red zone of **A** and **F**) or sub-fractions thereof with low LIP (above the blue line) or high LIP level (below the blue line) were analyzed. **K.** Percent thymocytes with polarized mitochondria with a high LIP in total T cells or T-cell subsets of *Fth^lox/lox^* (*white*) and *Fth* ^Δ/Δ^ mice (*grey*). **L.** Percent cells with a low TMRM fluorescence indicating depolarization in each subset of *Fth^lox/lox^* (w*hite*) and *Fth* ^Δ/Δ^ mice (*grey*). **M.** Graphical representation of all subset data obtained in **C–E** and **H–J**. T cells in each subset expressed as % of T cells with polarized mitochondria in the low LIP (*white*), total (*medium grey*) or high LIP (*dark grey*) fraction of *Fth^lox/lox^* and *Fth* ^Δ/Δ^ mice. Subsets for each color and separate genotype add up to 100%. Results are average values of 7 or 8 mice ± SD. ***p<0.0005; **p<0.005; *p<0.05.

Parallel to B cells we analyzed thymocytes of the same mice for the LIP and mitochondrial depolarization (Fig. 3). Thymocytes of both *Fth^lox/lox^* and *Fth* ^Δ/Δ^ mice showed distinct frequencies of high-LIP cells (Fig. 3A,F), that could be unquenched by chelation with deferiprone (Fig. 3B,G). Both *Fth^lox/lox^* and *Fth* ^Δ/Δ^ mice showed some cells with complete mitochondrial depolarization unable to shift in calcein fluorescence with chelation. They were considered as dead and not included in the subset analysis. All thymocyte subsets of *Fth* ^Δ/Δ^ mice showed a 3- to 8-fold increased number of high-LIP cells compared to *Fth^lox/lox^* mice (Fig. 3K). Mitochondrial depolarization (Fig. 3A,F) was increased in all T cell subsets of *Fth* ^Δ/Δ^ compared to *Fth^lox/lox^* mice but reached significance only in the CD4 SP subset (Fig. 3L). Thymocytes with polarized mitochondria were further analyzed for T cell subsets, either in total or separated into low and high LIP sub-fractions (Fig. 3C–E,H–J,M). They showed a high LIP-dependent selection against the mature CD4 and CD8 SP as well as the DN subsets independent of the deletion (Fig. 3M).

### B-cell Specific CD19-Cre Mediated Fth Deletion Confirms Reduction in Mature B Cells

The poly-IC induced deletion by Mx-Cre leads to a complex phenotype as the Mx-promoter responds to interferon most effectively in the liver and bone marrow, but also several other tissues with lower efficiency [9], [26]. To verify that Mx-Cre mediated effects were cell autonomous, we crossed *Fth^lox/lox^* mice with transgenic CD19-Cre mice to induce the Fth deletion in the earliest recognizable B-lineage cells during development [28], [40]. In conjunction we made the mice homozygous for the Rosa-EYFP allele that serves as an indicator of active Cre recombination [29]. Enhanced yellow fluorescent protein (EYFP) is expressed from Rosa-EYFP when Cre removes intervening loxP-flanked stop codons. As controls, we bred Fth*^+/+^* mice homozygous for Rosa-EYFP and heterozygous for CD19-Cre.

**Figure 4:**
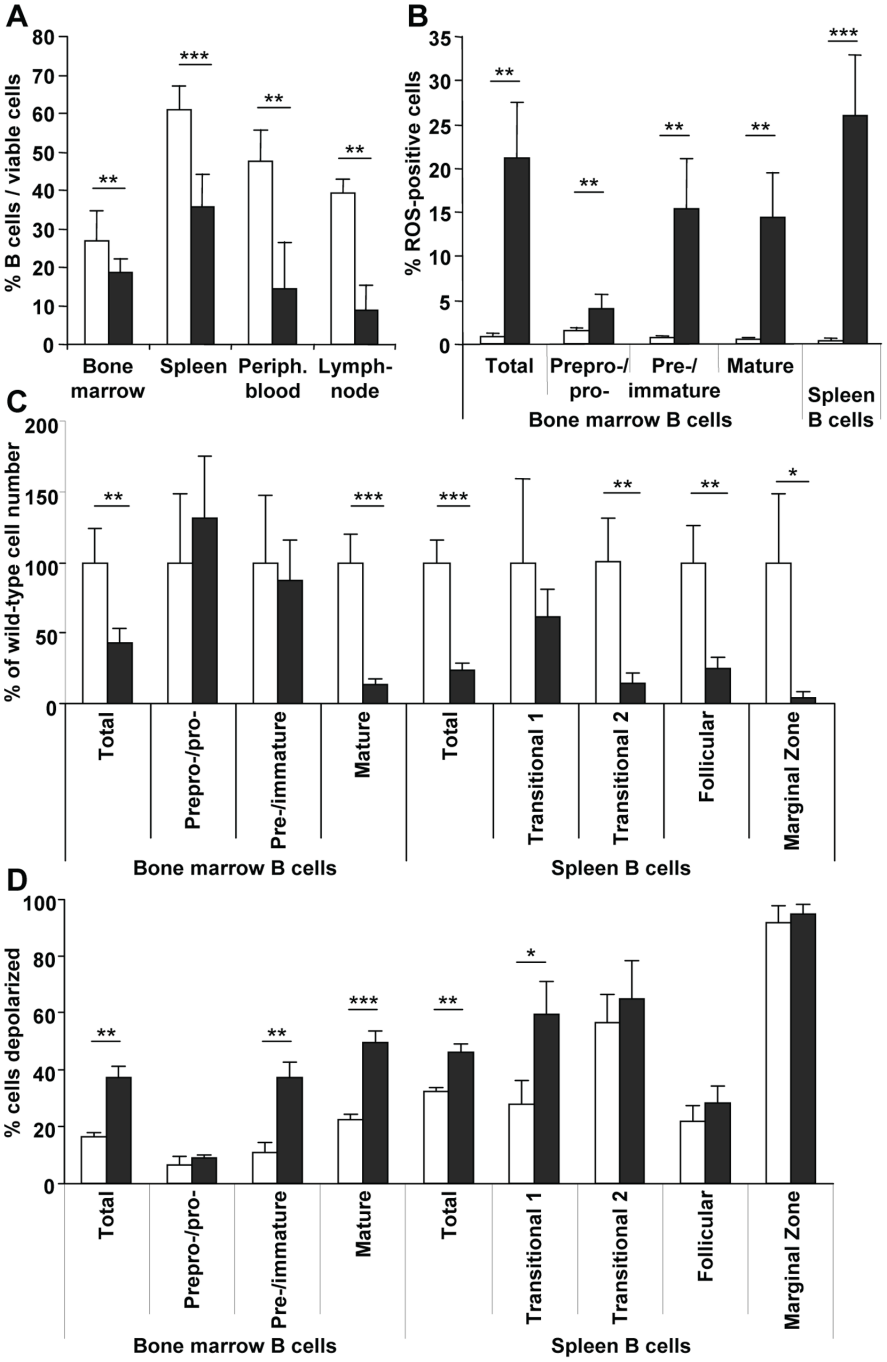
Reduced mature B-cell number in mice with a B-cell specific Fth deletion. *Fth^+/+^*;CD19-Cre*^+^* (*white*) and *Fth* ^Δ/Δ^ (*grey*) mice carried the CD19-Cre allele for B-cell specific deletion and Rosa-EYFP allele as a marker for cells where CD19-Cre is active. B cells were in all instances identified as B220^+^, CD19^+^ and EYFP^+^. **A.** Viable B cells among total cells were determined in different lymphoid tissues. **B.** B cells were stained with dihydroethidium to determine the % cells with ROS activity above background. **C.** Bone marrow B cells were divided into 3 subsets with antibodies against CD93 and CD43 as shown in Fig. 2B: CD93^+^/CD43^+^, prepro−/pro-; CD93^+^/CD43^−^, pre−/immature; and CD93^−^/CD43^−^, mature B cells. Spleen B cells were divided into 4 subsets with antibodies against CD21, CD23, and IgM, separating transitional stages 1 and 2, follicular, and marginal zone B cells. **D.** Staining with TMRM was used to determine mitochondrial depolarization for each subset. Values represent the % of the parent population (gating as shown in Fig. 2A). Results are average values of 8 mice ± SD. ***p<0.0005; **p<0.005; *p<0.05.

The frequency of B cells was strongly diminished in all lymphoid tissues of *Fth* ^Δ/Δ^ mice compared to control mice (Fig. 4A). EYFP^+^ B cells were further characterized according to subsets (Fig. 4C). The reduction in the bone marrow was entirely due to fewer mature B cells. The subset distribution in EYFP^+^ cells was similar to that obtained in high-LIP B cells after Mx-Cre mediated deletion (not shown). CD21, CD23, and IgM were used to define transitional stages 1 and 2, follicular and marginal zone B cells in the spleen [41]. There was a significant reduction in *Fth* ^Δ/Δ^ mice beginning at the transitional stage 2 (Fig. 4C). Mitochondrial depolarization was increased by the deletion beginning at the pre−/immature stage in the bone marrow and transitional stage 1 in the spleen (Fig. 4D). It was associated with a marked ROS production (Fig. 4B).

**Figure 5:**
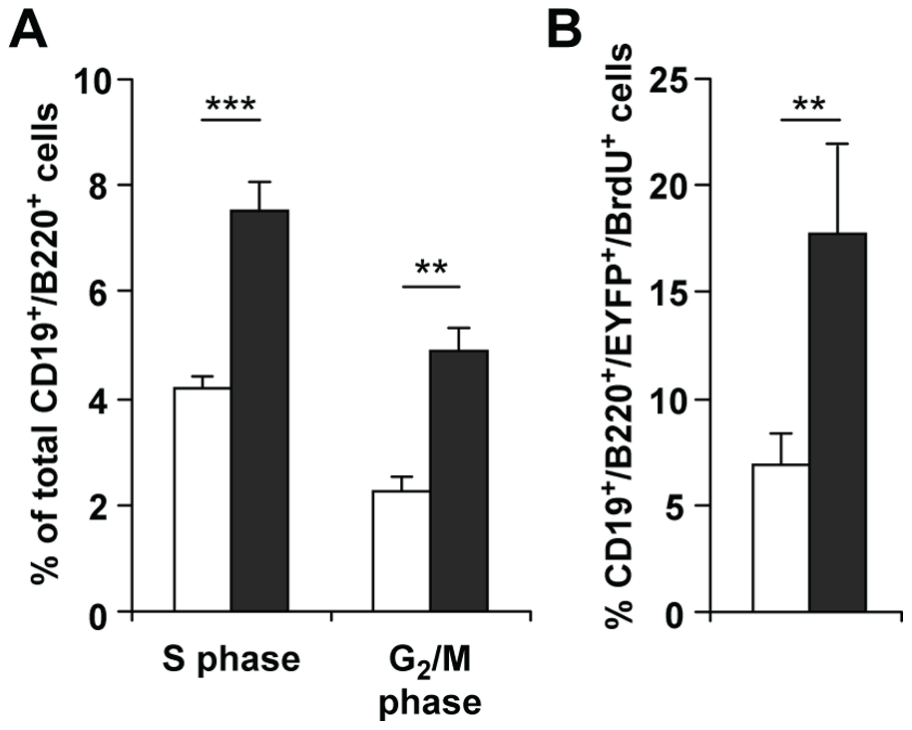
Proliferation and cell division of B cells in bone marrow of mice with CD19-Cre mediated Fth deletion. Cells of *Fth^+/+^*;CD19-Cre*^+^* (*white*) and *Fth* ^Δ/Δ^ (*grey*) mice were identified as B220^+^, CD19^+^ and EYFP^+^. **A.** Cells in S and G_2_/M phases of the cell cycle were analyzed by FACS. **B.** Mice were exposed to BrdU *in vivo* for 12 h prior to the isolation of bone marrow B cells. BrdU was detected by FACS. Results are average values of 3 mice ± SD. ***p<0.0005; **p<0.005.

As ferritin stores iron, which is essential for B cell proliferation, we analyzed whether the Fth deletion had an effect on B cell proliferation by examining the number of cells that enter the cell cycle during development. The number of bone marrow B cells in S and G_2_/M phase was increased by about 3% in *Fth* ^Δ/Δ^ with respect to *Fth^+/+^*;CD19-Cre mice (Fig. 5A) indicating an increased frequency of cell division. This was associated with an enhanced BrdU incorporation in *Fth* ^Δ/Δ^ B cells, independent of EYFP expression (Fig. 5B). These observations reflect the imbalance in B-cell homeostasis due to the Fth deletion and show that the reduction of B-cell numbers is not due to a block in cell division.

### Splenic B-cell Survival *in vitro* Requires Fth

**Figure 6:**
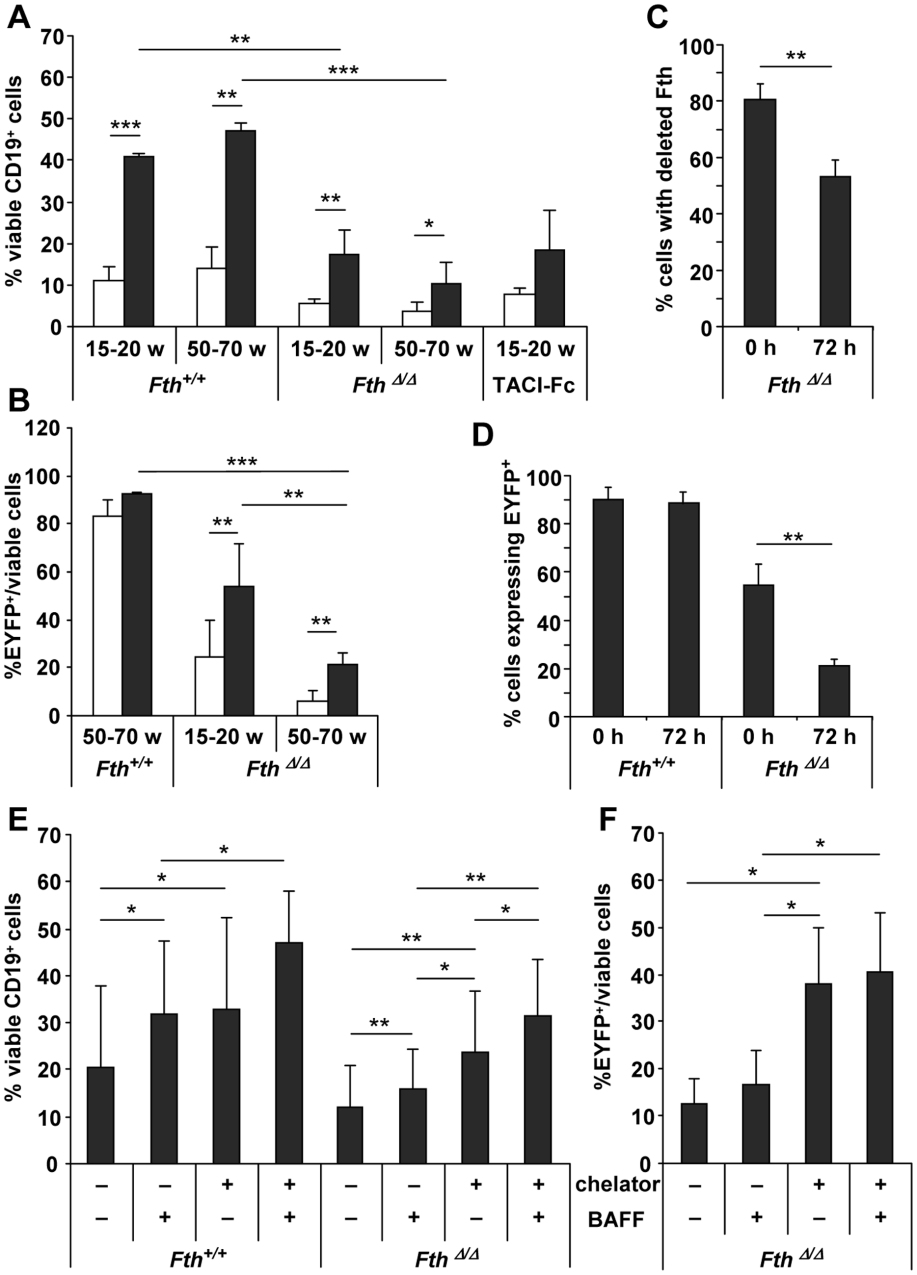
Fth deletion blocks BAFF-supported survival of spleen B cells *in vitro*. B cells were isolated from either *Fth^lox/lox^* and *Fth* ^Δ/Δ^ mice at 15–20 weeks (w) or CD19-Cre*^+^*;*Fth^+/+^* and *Fth* ^Δ/Δ^ mice at 50–70 w, and compared in their response to BAFF. For this, CD19^+^ splenocytes were separated on magnetic beads and cultured *in vitro* in absence (*white*) or presence (*grey*) of BAFF (20 ng/ml) for 72 h. **A.** Cell viability was determined by FACS based on scatter 72 h after BAFF addition and expressed as % survival compared to plated cells. The additional strain secreting TACI-Fc blocking BAFF was used as a negative control for BAFF regulation and analyzed in duplicate. **B.** EYFP^+^ B cells in the viable cell population (**A**) were measured 72 h after BAFF addition and plotted as % EYFP^+^ among viable B cells. **C.** % of viable CD19^+^ cells that harbor the Fth deletion as determined by genomic PCR at time 0 h and 72 h of cell culture. **D.** % of viable CD19^+^ B cells from CD19-Cre*^+^*;Rosa-EYFP;*Fth^+/+^* and CD19-Cre*^+^*;Rosa-EYFP;*Fth* ^Δ/Δ^ that are EYFP^+^ at time 0 h and 72 h of cell culture. **E.** Viability of CD19^+^ B cells in absence or presence of iron chelator deferiprone and BAFF after 24 h of culture. **F.** Viability of 15–20 w old EYFP^+^ B cells in absence or presence of 300 µM deferiprone and BAFF (20 ng/ml) after 24 h of culture. In experiments A, B, E, and F, the 15–20 w old control mice were *Fth^lox/lox^* littermates without CD19-Cre, while the 50–70 w old control mice had a *Fth^+/+^*;CD19-Cre*^+^* genotype. All cell cultures were analyzed in duplicates. Results are average values of 3 to 5 independent experiments ± SD. ***p<0.0005; **p<0.005; *p<0.05.

To further characterize the defect in mature B-cells observed *in vivo*, CD19^+^ spleen cells were cultured *in vitro* to assay B-cell survival in the presence of BAFF. This factor is necessary and sufficient for B-cell survival both *in vivo* and *in vitro* by activating NF-κB2-mediated transcription of several intracellular proteins that contribute to cell survival [41]. Cell survival was measured after 3 days in culture based on cell size and shape using forward and side scatter in flow cytometry. Independently of the genotype, BAFF addition increased viability approximately 3-fold with respect to untreated control cells (Fig. 6A). The assay demonstrated that Fth was essential for B-cell survival. BAFF-mediated 3-day survival was reduced from 41% in *Fth^+/+^* to 17% in *Fth* ^Δ/Δ^ B cells of mice aged 15–20 weeks. This reduction was similar to the one observed with B cells of TACI:Fc mice transgenic for a secreted BAFF receptor, which acts as a dominant negative BAFF inhibitor [41]. With B cells of mice aged 50–70 weeks, the BAFF-mediated survival was similar to the 15–20 week group, and only slightly but not significantly lower for *Fth* ^Δ/Δ^ B cells (Fig. 6A). This difference was, however, significant for *Fth* ^Δ/Δ^ *;*EYFP^+^ B cells, the survival of which diminished from 54% at 15–20 weeks to 18% at 50–70 weeks (Fig. 6B). In control B cells, it was 2- to 5-fold higher (Fig. 6B) and unaffected by age (not shown). Thus, BAFF supports the survival of both deleted and undeleted cells, but the number of surviving Fth-deleted EYFP^+^ cells is lower and reduced further with age, indicating selection against the Fth recombination. This conclusion was supported by measuring the frequency of genomic Fth deletion and EYFP^+^ at 0 h and 72 h cell culture in the 50–70 week age group. At time 0 h, 81% of the viable CD19-Cre^+^ cells showed the Fth genomic deletion (Fig. 6C) and 54% were EYFP^+^ (Fig. 6D). At 72 h, the frequency of the genomic deletion was reduced to 53% and that of EYFP^+^ cells to 21%. Thus, the loss of approximately 30% B cells with the Fth genomic deletion and 30% EYFP^+^ B cells occurred concomitantly during the *in vitro* cell culture rather than *in vivo*. No negative selection against wild-type EYFP^+^ B cells was observed (Fig. 6D).

The importance of Fth in B cell survival was further examined by addition of the iron chelator deferiprone (Fig. 6E). After 24 h of chelation, both BAFF-mediated and BAFF-independent viability was increased about two fold regardless of the Fth deletion. The effect of the chelator and BAFF together was additive. That chelation increased survival independently of the deletion might imply that Fth was needed for survival independently of chelatable iron in the LIP. However, chelation effectively blocked the selection against EYFP^+^ cells independent of the presence of BAFF, with a 3-fold increase in the viable fraction to more than 40%, almost the value obtained at the start of the experiment (Fig. 6F). Therefore, selection against survival after the Fth deletion is due to an increase of the LIP that can be rescued by addition of a chelator.

### Specific Deletion of Fth by CD4-Cre Reduces Cell Number of Primary and Peripheral T-Cell Subsets

**Figure 7:**
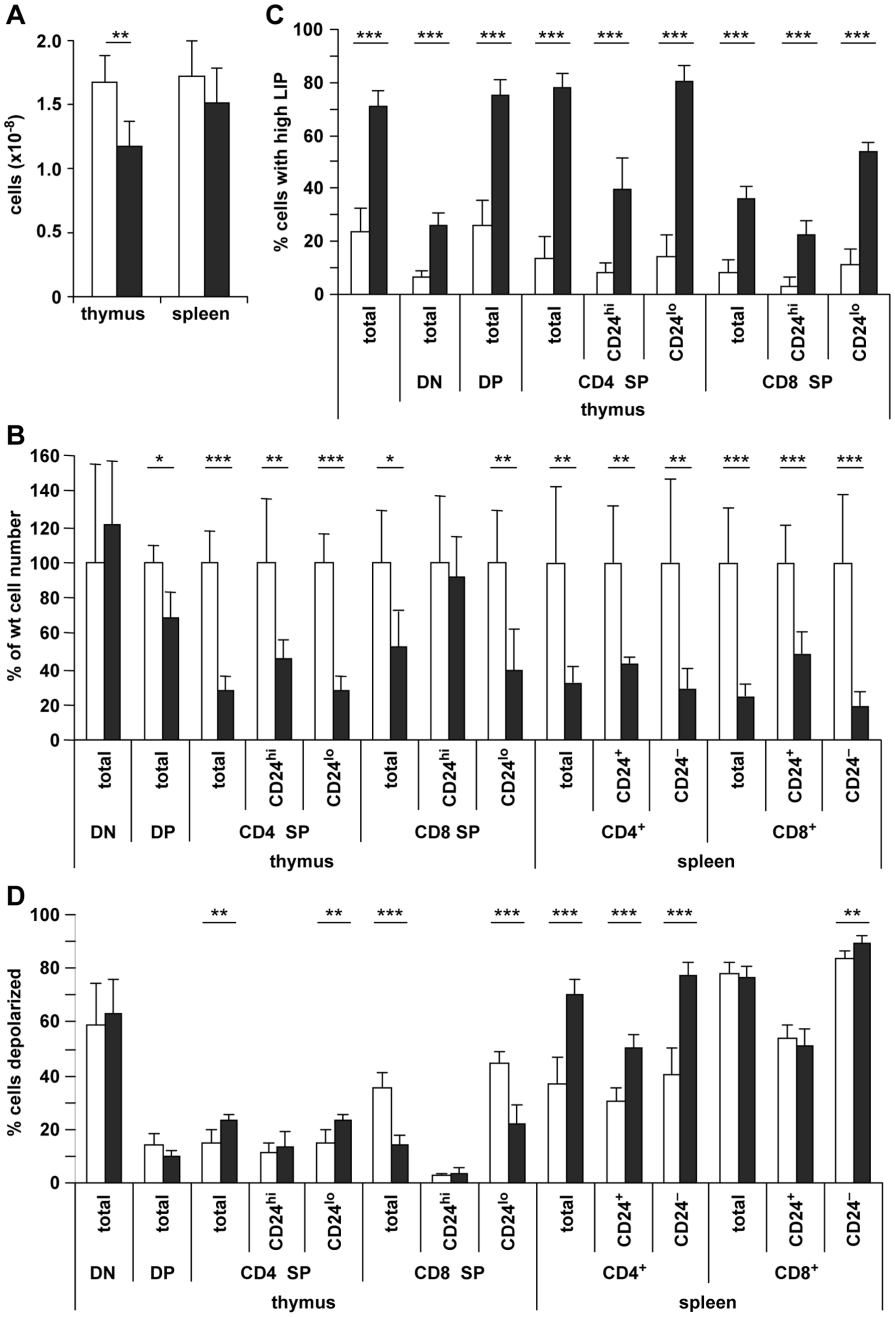
CD4-Cre mediated Fth deletion induces a reduction of T cells in thymus and spleen concomitant with high LIP and mitochondrial depolarization. Lymphocytes of thymus and spleen of 5–7 weeks old *Fth^+/+^*;CD4-Cre*^+^* or *Fth^lox/lox^* control mice (*white*) and *Fth* ^Δ/Δ^ mice (*grey*) were stained with calcein AM and TMRM, and in addition with anti-CD4 and anti-CD8α antibodies as detailed in Fig. 3. They were further separated into high- and low-level CD24 expressing cells. No significant differences were visible between 3 *Fth^+/+^*;CD4-Cre mice and 3 *Fth^lox/lox^* control mice, and data were pooled. **A.** Total viable cell number in thymus and spleen. **B.** Number of cells in T cell subsets in thymus and spleen of Fth^Δ/Δ^ mice relative to control mice, set as 100%. **C.** % cells with low calcein staining due to quenching by high LIP. **D.** % cells with low TMRM staining that is a sign of mitochondrial depolarization. Results are average values of 6 mice ± SD. ***p<0.0005; **p<0.005; *p<0.05.

In order to verify the observations on T cells made with Mx-Cre induced *Fth* ^Δ/Δ^ mice, we crossed *Fth^lox/lox^* mice with CD4-Cre mice. CD4-Cre initiates recombination as early as the DN3-TCRβ^+^ stage of T-cell development [42] and is complete in the DN4 and DP thymocytes [27], [42]. T cells were analyzed in the thymus and spleen at adulthood with respect to subsets and maturation. The rate of the Fth genomic DNA deletion in thymocytes of *Fth^lox/lox^*;CD4-Cre mice was 98±1% (n = 6). *Fth* ^Δ/Δ^ mice showed a significantly reduced cell number in the thymus, and a decrease in the spleen that was insignificant (Fig. 7A). Analysis of thymocytes showed that DN cells were unaffected, whereas CD4^+^CD8^+^ DP, and CD4 SP and to a slightly lesser extent CD8 SP cells were reduced in Fth^Δ/Δ^ compared to control mice (Fig. 7B). Analysis of CD24, which is lost with maturation, was used to further distinguish immature and mature SP subsets. Mature SP subsets with low CD24 were reduced more than the less mature SP subsets with high CD24. There was a similar reduction of T cells in the periphery as measured in the spleen (Fig. 7B). The mediated Fth deletion strongly increased the LIP in all subsets of thymocytes (Fig. 7C). This increase was stronger for mature (CD24^low^) than immature CD4 SP and CD8 SP thymocytes. In the spleen, a slight LIP increase could be detected in CD24^−^ cells but none in CD24^+^ T cells (not shown). The increase in LIP correlated with a slight decrease in TfR1 expression (not shown). The loss of CD4 SP thymocytes and CD4^+^ splenocytes in *Fth* ^Δ/Δ^ mice was associated with increased depolarization relative to cells from control mice (Fig. 7D). In contrast, CD8 SP cells in *Fth* ^Δ/Δ^ thymus showed a decrease of mature CD24^low^ cells with depolarized mitochondria, while CD8^+^ T cells in spleen showed no change. The results for these two subsets are qualitatively similar to those in the Mx-Cre induced deletion (Fig. 3L). Yet with good reproducibility, DN thymocytes and spleen CD8^+^ cells showed a low TMRM fluorescence and hence scored a high depolarization rate, even in control mice (Fig. 7D). This might be due to a poor absorption of the dye in these cells and should not be over-interpreted, as deleted and control mice showed no differences. Cell death as measured by uptake of 7AAD occurred in the CD24^−^ subset of the spleen but not the thymus (not shown).

## Discussion

The present study shows that an Fth deletion in hematopoietic cell compartments reduced the number of B and T lymphocytes, while other cell lineages like granulocytes, monocytes and nucleated erythrocytes were not affected (Fig. 1). For B cells, the results were similar in mice deleted by Mx-Cre compared to the specific deletion with CD19-Cre (Figs. 1, 2, 4), demonstrating that the reduced development or survival was cell autonomous. Also for T cells, both Mx-Cre and CD4-Cre mice showed reduced T-cell subsets. This reduction was broader for the Mx-Cre induced deletion than the CD4-Cre mediated deletion (Figs. 1, 3, 7), possibly because CD4 is activated later in T-cell maturation. The analysis of relative subset frequencies indicates that the reduction in cell development or survival after the Fth deletion was the consequence of an increased LIP. It highlights that Fth and its ferroxidase activity are absolutely required for iron storage and scavenging of excess labile iron to protect cells from oxidative damage [8], [9]. Yet, a high LIP alone may not be sufficient for cell death to occur as the overall reduction in B and T cell numbers was in the range of 30% (Figs. 1, 4, 7) while we observed rates of high LIP in the range of 50% or above (Figs. 2, 3, 7). Cell death appears to involve in addition the depolarization of mitochondria in B and T cells with a high LIP. The depolarization, associated with ROS production, starting at the pre−/immature stage in B cells (Fig. 4B) is a characteristic of proton pump uncoupling that increases superoxide production [43]. In T cells, increased depolarization was detected in Mx-Cre deleted thymus already at the DN stage but was significant only at the CD4 SP stage (Fig. 3) while in CD4-Cre deleted thymus and spleen again only the CD4 SP stage cells were significantly different from control cells (Fig. 7). No increase in ROS activity was detectable (not shown) possibly due to the very rapid elimination of apoptotic T cells.

The absence of intracellular iron stores did not inhibit cell proliferation as overall B-cell proliferation was actually stimulated by the Fth deletion in conjunction with the loss of mature B cells (Fig. 5). It suggests that proliferating cells had a sufficient amount of iron available for protein synthesis. This finding is not necessarily in contradiction with previous studies concluding that intracellular iron stores of ferritin are needed to support the more massive cell proliferation in the context of antigen or mitogen stimulation [11]. Here, the augmented LIP following Fth deletion (Figs. 2, 3, 7) could potentially increase iron availability for proliferation although leading ultimately to cell death. During cell proliferation cells might use up excess iron for *de novo* enzyme biosynthesis, while fully differentiated resting cells are expected to have less biosynthetic activity and would, therefore, be more exposed to excess iron and ROS. This might explain why mainly mature B cells are affected by the Fth deletion. Similarly, for T cells, immature cells undergo expansion through cell divisions, while those which complete their maturation become resting prior to antigen stimulation [44]. Thus, increased proliferation at immature SP stages with a characteristic high CD24 expression [45], [46], in both the thymus and periphery [16], could explain their resistance to excess LIP compared to mature SP stages (Fig. 7).

The *in vitro* B cell culture assays with BAFF provide evidence that Fth is required for the survival of B cells (Fig. 6). The equivalent effect on survival by the BAFF negative inhibitor protein TACI-Fc [30] compared to the Fth deletion suggested Fth may be induced by BAFF as reported previously [12]. However, Fth transcription was unaffected by BAFF (not shown). As iron chelation almost compensated the negative effect of the Fth deletion, it appears that keeping the LIP in check by iron storage reduces the extent of ROS and cell damage [8], [9], [12], [47]. We conclude that Fth and BAFF are both independently required for optimal B-cell survival.

The presence of a high LIP in T cells of Fth wild-type mice associated with depolarization suggests such a process may be operable in normal T cell (Fig. 3). This would explain why the selection against mature high-LIP cells was as important in wild-type as in deleted mice (Fig. 3M). NF-κB induces Fth transcription to prevent ROS formation and cell death in 3T3 fibroblasts [12], and it may similarly be protective in lymphocytes. After deletion of Fth, lymphocytes would loose this protection, and the high LIP might shift the normal sequence of clonal selection towards increased apoptosis. This hypothesis remains somewhat speculative. In the Fth-deleted mice with Mx-Cre we rather conclude that the loss of T cells occurs at an early developmental stage prior to clonal selection (Fig. 1). For the Fth deletion in B cells, or T cells by CD4-Cre, where we observe negative selection at rather late stages, close to terminal differentiation, the question remains open. It is noteworthy that *TfR1* ^Δ/Δ^ mice with a perturbed iron metabolism also show severe changes in lymphocyte survival [21]. There, thymocytes did not differentiate beyond the early DN stage and B cells did not reach full maturity, a phenotype that looks similar to the one described here. The authors conclude that the supply of extracellular transferrin iron is probably required at lymphocyte differentiation stages where cells proliferate. Both knock-out models highlight the fact that lymphocyte development is sensitive to an appropriate LIP. Our observations may provide a basis to understand alterations of T-cell levels in human patients with iron-overload that have potentially an increased LIP [22], [23], [24], [25]. While the present study focused on mechanisms that diminish lymphocyte numbers, further work should be devoted to possible effects on the immune defense of our Fth deleted mice.
